# Fractional amplitude of low-frequency fluctuations during music-evoked autobiographical memories in neurotypical older adults

**DOI:** 10.3389/fnins.2024.1479150

**Published:** 2025-01-23

**Authors:** Teresa Lesiuk, Kaitlyn Dillon, Giulia Ripani, Ioannis Iliadis, Gabriel Perez, Bonnie Levin, Xiaoyan Sun, Roger McIntosh

**Affiliations:** ^1^Department of Music Therapy, Frost School of Music, University of Miami, Coral Gables, FL, United States; ^2^Department of Psychology, University of Miami, Coral Gables, FL, United States; ^3^Department of Neurology, Miller School of Medicine, University of Miami, Miami, FL, United States

**Keywords:** music evoked autobiographical memory, fALFF, parahippocampal, LIWC features, older adults

## Abstract

**Introduction:**

Researchers have shown that music-evoked autobiographical memories (MEAMs) can stimulate long-term memory mechanisms while requiring little retrieval effort and may therefore be used in promising non-pharmacological interventions to mitigate memory deficits. Despite an increasing number of studies on MEAMs, few researchers have explored how MEAMs are bound in the brain.

**Methods:**

In the current study activation indexed by fractional amplitude of low frequency fluctuations (fALFF) during familiar and unfamiliar MEAM retrieval was compared in a sample of 24 healthy older adults. Additionally, we aimed to investigate the impact of age-related gray matter volume (GMV) reduction in key regions associated with MEAM-related activation. In addition to a T1 structural scan, neuroimaging data were collected while participants listened to familiar music (MEAM retrieval) versus unfamiliar music.

**Results:**

When listening to familiar compared to unfamiliar music, greater fALFF activation patterns were observed in the right parahippocampal gyrus, controlling for age and GMV. The current findings for the familiar (MEAM) condition have implications for cognitive aging as persons experiencing age-related memory decline are particularly susceptible to volumetric reduction in the parahippocampal cortex. *Post-hoc* analyses to explore correlations between brain activity and the content of MEAMs were performed using the text analysis program Linguistic Inquiry and Word Count.

**Discussion:**

Our findings suggest that MEAM-related activation of the parahippocampal cortex is evident in normative older adults. However, it is yet to be determined whether such brain states are attainable in older adult populations diagnosed with mild cognitive impairment and/or prodromal Alzheimer’s disease.

## 1 Introduction

Music and memory are deeply intertwined as listening to music can trigger memories of past experiences (Belfi et al., 2015; Jakubowski and Ghosh, 2021; Janata et al., 2007; Schulkind et al., 1999). These memories, known as music-evoked autobiographical memories (MEAMs; Janata et al., 2007), have received increasing attention within the context of social and behavioral sciences, as well as clinical and neurological research. Researchers in the field have shown that MEAMs can stimulate long-term memory mechanisms while requiring little retrieval effort (El Haj et al., 2012; Jakubowski and Ghosh, 2021; Janata, 2009). MEAMs can therefore be used in promising non-pharmacological interventions to mitigate memory deficits (Kaiser and Berntsen, 2023).

Within the context of social and behavioral sciences, scholars have focused on the cognitive and emotional aspects of MEAMs, while addressing the conditions that prompt their emergence. MEAMs include associations with autobiographical knowledge, such as references to persons, places, lifetime periods, and specific events (i.e., *nature* or *cognitive content*; Janata et al., 2007). Although music-evoked autobiographical associations are common phenomena across adulthood, researchers have documented age-related differences that may stem from changing internal drives (Lesiuk and Ripani, 2025). For instance, young adults more often recall persons or relationships as they may be mainly focused on building careers and relationships (Janata et al., 2007). In contrast, older adults tend to associate music with different life periods, as they may be engaging in retrospective reflections about their lives (Lesiuk and Ripani, 2025).

Across age levels, MEAMs are likely to be associated with positive and mixed emotions, such as happiness, youthfulness, and nostalgia (i.e., *emotional content*; Jakubowski and Ghosh, 2021; Janata et al., 2007; Juslin and Vastfjall, 2008). In addition, MEAMs can occur in a variety of contexts. Although researchers have mainly addressed the relationship between music and memories in laboratory settings (Belfi et al., 2015; Janata, 2009; Janata et al., 2007; Zator and Katz, 2017), MEAMs can also occur in naturalistic environments during routine tasks and moments of relaxation (Jakubowski and Ghosh, 2021).

Regardless of the setting, music-evoked memories are likely to be triggered by pieces that are autobiographically salient and emotionally charged (Bartlett and Snelus, 1980; Schulkind et al., 1999). For instance, older adults tend to recall autobiographical memories when listening to music from their adolescence and young adulthood (Krumhansl and Zupnick, 2013; Zimprich and Wolf, 2016), while young adults’ mental imagery is triggered by pieces that were popular during their childhood (Janata et al., 2007). One reason for this phenomenon is that listening to autobiographically salient music often evokes a “feeling of knowing,” which facilitates the recollection of memories (Jäncke, 2008). Furthermore, memories are not stored in isolation but are intricately linked with emotional states (Bower, 1981). As music evokes strong emotions, it can be involved in forming long-term memories (Jäncke, 2008). Listening to emotionally charged music can then serve as an effective stimulus for memory retrieval (Eschrich et al., 2008). Of note, the term “autobiographical music” is used interchangeably with familiar music, and the term “non-autobiographical music” is used interchangeably with unfamiliar music in this study.

Despite the growing body of research on MEAMs, the specific neural mechanisms underlying these memories remain relatively underexplored (Janata, 2009; Wilkins et al., 2014). Although limited in number, previous studies have highlighted the role of the default mode network (DMN) and its components, such as the medial prefrontal cortex (mPFC) and posterior cingulate cortex (PCC), which are crucial for self-reflective thought and socio-emotional memory (Kizilirmak et al., 2023). Wilkins et al. (2014) documented that the DMN showed the highest connectivity when participants listened to their preferred music. Similarly, Janata (2009) investigated the integration of music and autobiographical memories in the mPFC, demonstrating that the dorsal mPFC was activated during emotionally salient autobiographical songs.

Expanding on these findings, researchers have also explored the role of other brain regions, such as the parahippocampal cortex, which interact with the DMN during memory-related cognitive processes and subserve higher-order cognitive and affective functions (Aminoff and Tarr, 2015). For instance, Leaver et al. (2009) conducted a seminal study wherein young adult participants listened to the final 32 s of either highly familiar or unfamiliar instrumental recordings. They observed significantly greater activity in the PCC and the left parahippocampal cortex during the familiar listening condition. Satoh et al. (2006) also found that when participants judged the familiarity of musical pieces, there were greater changes in regional cerebral blood flow throughout the medial temporal lobe and the parahippocampal cortex.

The parahippocampal cortex has been strongly implicated in fMRI studies involving MEAMs. For example, the search for and retrieval of autobiographical memories evoked by music were associated with activation in the parahippocampus, hippocampus, left inferior temporal gyrus, and left PCC (Ford et al., 2011). In another study, autobiographical memory recollection during familiar music listening involved a cortico-ponto-cerebellar network including the left precuneus, bilateral anterior cingulum, parahippocampal gyri, frontal inferior operculum, ventral anterior insula, frontal medial orbital gyri, and parts of the cerebellum (Falcon et al., 2022).

While these studies have provided insights into how different areas are active during music listening and MEAM recall, there is still a need to clarify the specific brain regions that are involved during the process of MEAM retrieval and to identify differences in relation to the content of autobiographical memories. In addition, these studies mainly implemented a resting-state paradigm to study patterns of coactivation during what may be considered a functional task, i.e., autobiographical memory retrieval. A central issue in utilizing fMRI connectivity metrics as diagnostic biomarkers of behavioral performance and neuropathology is the reduced reliability from the task-based brain activity incurred before or during the resting-state acquisition, which can corrupt the “resting” functional connectivity signal (Shah et al., 2016).

One approach to circumvent the inherent assumptions and dependencies of traditional functional connectivity analyses is to capture the blood-oxygen-level-dependent (BOLD) signals evoked during active listening tasks by quantifying the amplitude of low-frequency fluctuations (ALFFs). The fractional amplitude of low-frequency fluctuations (fALFFs) measures the relative contribution of low-frequency fluctuations within a frequency band (0.01–0.08 Hz) and is thought to reflect a different aspect of the BOLD signal more sensitive to the amplitude of regional neuronal activity (Egorova et al., 2017). Whereas the BOLD signal extracted from resting-state connectivity analyses reflects the temporal synchrony between distinct regions, fALFF captures the amplitude of spontaneous neuronal activity and may even identify regions showing aberrant activity in cognitively impaired older adults (Zou et al., 2008). In particular, fALFF measures the relative contribution of low-frequency fluctuations within a specific frequency band relative to the whole detectable frequency range, thereby allowing researchers to characterize the amplitude of spontaneous neuronal activity in hippocampal regions germane to the pathophysiology of neuropsychiatric disorders such as dementia and milder forms of cognitive impairment (Chen et al., 2024; Yang et al., 2018; Zhou et al., 2020).

Researchers suggest that the spontaneous activity indexed by ALFF is not just present during the resting state but also during and after behavioral task performance (Fox et al., 2006; Fair et al., 2007). Thus, ALFF responses measured during task paradigms represent a combination of spontaneous activity and stimulus-evoked responses. While resting-state connectivity analyses allow for elucidating correlative patterns of spontaneous activity, ALFF indices are spatially unconstrained measures that can be collected independent of assumptions concomitant with *a priori* seed and network designation (Li et al., 2012; Montalà-Flaquer et al., 2023). Furthermore, although block designs have been the convention for music listening paradigms, the continuous quantification of low-frequency fluctuations is becoming more common in the field as an uninterrupted measure of brain responses to naturalistic stimuli shows greater reliability than when assessed by block design where auditory stimuli are separated by silence or non-musical sound (LI et al., 2012; Maguire, 2012; Sonkusare et al., 2019). Furthermore, ALFF responses to music have most recently been implemented in therapeutic applications due to the reliability of the signal from session to session (Wall et al., 2023). Thus, given our group’s interest in the potential implications of MEAMs in a therapeutic setting, the use of fALFF allows us to circumvent some of the spatial, temporal, and ecological limitations of the standard resting-state and task-based fMRI methodological approaches.

Given that music listening is associated with the spontaneous production of thoughts, feelings, and emotions, a technique developed to assess global changes in this spontaneity is perhaps better suited for the MEAM paradigm than traditional block designs or resting-state analyses (Di Liberto et al., 2021). Most recently, the mean ALFF (mALFF)/fALFF approach has been implemented with the MEAM paradigm. Carrière et al. (2020) used five excerpts of preferred musical pieces selected by eight healthy young-to-middle-aged adults and by the loved ones of nine post-comatose patients. Compared to resting state, listening to music was associated with increased ALFF in several brain regions including the brain stem, vermis, temporal pole, left inferior and middle temporal gyrus, left insular cortex, and left angular gyrus. Regarding fALFF, this study found no consciousness-level-dependent increases. Although the increased ALFF observed in this study implicates brain regions involved in music and auditory processing and stimulus recognition, the absence of an effect in the higher signal-producing fALFF analyses may suggest that these findings reflect greater noise than the BOLD signal.

Resting-state measures of ALFF are shown not only to predict task-evoked brain activity but also to correlate with cognitive-behavioral performance in adults (Liu et al., 2011; Wang et al., 2019; Yan et al., 2009; Zhao et al., 2014). More recently, investigations of healthy older adults confirmed that spontaneous brain oscillations in the parahippocampal gyrus are positively correlated with associative memory performance (Ren et al., 2015). Another study showed that older adults demonstrating superior self-initiated memory strategy use during an associative learning and memory task demonstrated predominant ALFF values in the superior frontal gyrus (Zheng et al., 2018). This coincides with the role of prefrontal cortices in strategic memory control processes (Shing et al., 2010).

Altogether, the MEAM paradigm has garnered attention from those seeking to understand how brain activity is differentially organized for familiar versus unfamiliar music. However, the interpretation of these findings presents challenges in reliably phenotyping brain activity during autobiographical retrieval. This is mainly due to the widely varying methodological approaches of capturing “resting-state” connectivity versus what may be argued as a task-based paradigm eliciting functional activity during memory retrieval. Moreover, given the importance of these memory processes in disorders of cognitive aging, it is imperative that these studies be reliably conducted in healthy older adults before we are able to extrapolate these findings to a clinical setting. Hence, the current study aimed to capture and compare whole-brain fALFF during familiar and unfamiliar MEAM retrieval in a sample of healthy older adults. Additionally, the current study aimed to investigate the impact of age-related gray matter volume (GMV) reduction in key regions influencing MEAM-related activation. As an additional, exploratory aim, this study sought to evaluate the associations between brain activity and the content of autobiographical memories. Meta-analytic and longitudinal studies suggest that volumetric reduction of hippocampal gray matter is associated with poorer memory performance on neuropsychological tests in healthy older adults (Barry et al., 2021; Chen et al., 2010; Van Petten, 2004). Moreover, age-related reduction in hippocampal volume is associated with decreased activation within the hippocampal and parahippocampal cortex during autobiographical memory retrieval (Maguire and Frith, 2003; Maguire et al., 2005; Viard et al., 2007).

## 2 Methods

### 2.1 Participants

This study involved 24 older adults residing in a major urban area in the Southeastern United States. The study was approved by the Institutional Research Board at the University of Miami. The sample consisted of 13 female (54.2%) and 11 male (45.8%) participants, with ages ranging between 60 and 80 years (*M* = 67.83, SD = 6.30). Participants exhibited typical cognitive aging, as assessed by the Montreal Cognitive Assessment tool (MoCA; Nasreddine et al., 2005; *M* = 28.50, SD = 1.73). No participant reported having hearing issues. The majority of participants identified themselves as white non-Hispanic (*n* = 13, 54.16%), while five participants (20.83%) described themselves as white Hispanic. Four participants (16.66%) selected the category African American, and two others (8.33%) preferred not to disclose their ethnicity.

Nearly half of the participants reported no prior musical training (*n* = 11, 45.8%). 1 participant (4.2%) was self-taught, 2 participants (8.4%) did not answer, and 10 participants (41.7%) received formal training, ranging from 1 to 6 years of music classes. Despite these differences, participants were actively engaged in musical activities. For example, many reported frequently listening to music during their childhood (*n* = 17, 70.9%) and adolescence/early adulthood (*n* = 20, 83.3%). On average, they estimated listening to music for 9.29 h per week (*SD* = 7.92). Regarding their attitude toward music, the majority of participants strongly agreed (*n* = 8, 33.3%), agreed (*n* = 5, 20.8%), and somewhat agreed (*n* = 2, 20.8%) with the statement “Music has played a significant role in my life.” Similarly, they mostly agreed (*n* = 11, 45.8%), strongly agreed (*n* = 5, 20.8%), and somewhat agreed (*n* = 5, 20.8%) that music evoked memories of events, people, and places.

### 2.2 Procedure

This study is the second phase of a larger research project, which unfolded in several steps (see Supplementary Figures S1, S2). In phase 1, participants completed an online questionnaire that included both open-ended and multiple-choice questions. Participants were asked to specify four autobiographical musical pieces, answer questions on their cognitive and emotional responses to self-selected music, and provide sociodemographic information.

In phase 2, participants were invited to our laboratory where a team of neurologists administered the Montreal Cognitive Assessment (MoCA; Nasreddine et al., 2005), a screening tool for detecting mild cognitive impairment (MCI). In addition, participants completed an fMRI scan that included a 12-min resting-state sequence, a T1 structural sequence (TR/TE = 2300/1 ms, FA = 9°, thickness = 1.0 mm, matrix size = 256 × 256), a 12-min autobiographical (familiar) music listening task, arterial spin labeling, a 12-min unfamiliar music listening task, and diffusion-weighted imaging. The familiar and unfamiliar music listening tasks were presented in a randomized order. Following the protocols approved by the institutional review board of a southern university, fMRI data were collected on a SIEMENS MAGNETOM 3.0 T XT Numaris/X VA50A-01NG (Standard Siemens headphones (e.g., Tim platform: mat.no. 10018373)) scanner using a 20-channel head coil. The fMRI protocol involved multiband acquisition to minimize scanner noise interference.

Both the autobiographical and non-autobiographical excerpts included three pieces. The non-autobiographical musical excerpt, the same for all participants, consisted of three pieces that are not part of common Western repertoires (i.e., Balinese Gamelan, Mongolian throat singing, and Zimbabwean Mbira music; Supplementary Figure S2). These pieces were selected to minimize familiarity and associations with the music, as recommended by Dr. Donald Hodges, music psychologist (personal communication). The autobiographical musical excerpt included three randomly selected pieces from the four self-selected pieces that participants indicated in phase 1. The research coordinator created MP3 files that included the non-autobiographical excerpt and a dedicated MP3 file with autobiographical music for each participant. Smooth transitions between pieces were achieved by gradually fading in and out. All MP3 files were preprocessed in the laboratory for loudness equalization to ensure consistent volume levels for the MR-audio stimulation system. Participants listened to both autobiographical and non-autobiographical music in the scanner using headphones, with the volume set equally across all conditions. Participants were asked to keep their eyes open, try not to move, listen and focus on the music, and experience any thoughts, memories, or emotions that might arise while listening to the music. The MRI technician verbally confirmed that the music clips were audible during the scan and no significant acoustic differences were observed between the familiar and unfamiliar music.

After the laboratory sessions, participants completed a second online questionnaire that included both open-ended and multiple-choice questions assessing cognitive and emotional responses to the musical excerpts that participants listened to during the brain imaging sessions. Finally, we conducted follow-up phone interviews. Responses to questionnaires 1 and 2, along with interview transcripts, were analyzed to characterize the cognitive and affective responses associated with the MEAMs during the listening tasks (Lesiuk and Ripani, 2024). Among these responses, answers to the question “If you would like to, briefly describe the event, person, place, or period of your life that this song/music reminds you of” were used for *post-hoc* analyses in this study.

### 2.3 Data analysis

Demographic data were analyzed using IBM SPSS 27.0 (IBM Corp, 2020). We installed the MIRToolbox in the MATLAB program to derive acoustic parameters of flatness, brightness, pulse clarity, key clarity, and loudness indicated by root mean square (RMS). These metrics were averaged across the three familiar music selections and compared to the parameters of the unfamiliar music selection. Familiar music was significantly different from unfamiliar music across all 5 indicators (Supplementary Tables S1a,b). The first-level functional brain images were preprocessed using Data Processing Assistant for Resting-State fMRI (DPARSF) V4.075, within the Data Processing & Analysis for Brain Imaging (DPABI) V2.0^1^, implemented in MATLAB R2015b (MathWorks, Natick, MA, USA). The second-level analyses were performed using the Statistical Parametric Mapping (SPM) 12 package.^2^

Initially, the MRI scanner removed the first five volumes to account for magnetization equilibrium effects. In total, 1,232 volumes were preprocessed from each participant. Functional images were not corrected for slice-timing differences due to the relatively fast multiband recording (TR = 0.575 s). Although participants exhibited small head movements (<2 mm), head movement artifacts were systematically identified and corrected by evaluating framewise displacement (FD), which was defined as the sum of the absolute derivative values of the six head realignment parameters (Power et al., 2012). The FD parameter values were later used in creating nuisance regressors in the individual-level analyses. The Friston 24-parameter model was used which included the six head motion parameters (three translations and three rotations) the 6 head motion parameters at the previous time point, and the 12 corresponding squared items as nuisance regressors; Friston et al., 1996). Within the general linear model used to estimate the linear trend of the BOLD signal, white matter and cerebrospinal fluid signals were regressed out from the fMRI time series. T1-weighted images were extracted prior to analysis using the fMRI Imaging of the Brain (FMRIB) software library’s (FSL) brain extraction tool algorithm. Subsequently, both the T1 anatomical and noise-corrected functional images were normalized to Montreal Neurological Institute space using the anatomical image-based unified segmentation–spatial normalization approach wherein the T1 anatomical image was co-registered to the mean of the functional images (Ashburner and Friston, 2005). Finally, the spatially normalized, noise-corrected functional images were resampled to a voxel size of 3 × 3 × 3 and smoothed with an isotopic Gaussian kernel of 6-mm full width at half maximum.

fALFF was computed using individual preprocessed data in order to quantify the regional intrinsic brain activities during the listening sessions (Zou et al., 2008). We define fALFF as the ratio between the sum of Fourier amplitudes within a specific low-frequency range (0.01–0.1 Hz) and the sum of Fourier amplitudes across the entire frequency range (0–0.2 Hz). fALFF was calculated for each voxel in the whole brain to create an fALFF map for each participant. These beta maps were entered into the group-level analysis for the rest, familiar, and unfamiliar listening conditions and further standardized by the global mean of the fALFF map in producing *Z*-scores. After fMRI preprocessing, individual fALFF maps were entered into a second-level analysis within SPM-12. The fMRI data reflecting the contrast between familiar and unfamiliar music listening conditions of all subjects were analyzed by multiple regression controlling for age and whole-brain GMV. The statistical threshold was set as *p* < 0.001 uncorrected, extent threshold: 20 voxels.

### 2.4 LIWC analysis

*Post-hoc* analyses to explore correlations between brain activity and the content of MEAMs were performed using the text analysis program Linguistic Inquiry and Word Count (LIWC) (Pennebaker, 2001). The LIWC program contains a dictionary of over 2,000 words in 70 language categories and counts the frequency of words in each category. The LIWC has been used to examine many types of written and oral language samples, and when paired with neuroimaging, it shows promise in elucidating the activation of specific areas associated with specific cognitive processes (Brook O’Donnell and Falk, 2015).

The analyses focused on (1) personal pronouns, (2) positive emotion words, and (3) negative emotion words. We used the LIWC2015 internal English dictionary. The first-person singular pronoun category included the words “I, me, and mine.” The positive emotion category consisted of 642 word forms and patterns (e.g., “beautiful,” “favorite,” “respect,” and “roman*”), while the negative emotion category includes 746 word forms and patterns (e.g., words such as “angry,” “jealous,” “sad,” and “unwelcome”). Patterns that end with a star (‘*’) match any word form that starts identically, i.e., the pattern “romance” matches both “romance” and “romances.”

Individual responses from the second online questionnaire (“If you would like to, briefly describe the event, person, place, or period of your life that this song/music reminds you of”) were imported into an Excel 2016 spreadsheet. Each individual response was associated with the participant. Spelling errors (which are attributable to the experimenter’s transcription) were corrected using Excel 2016’s built-in English spell-check system. One participant was excluded due to missing data. While the program allowed for the examination of all possible LIWC variables (Drives, Cognition, Affect, Social, Cultural, Lifestyle, Physical, and Perception), we selected semantic categories based on previous studies, suggesting that dimensions such as perception and cognition may be the most salient aspects of MEAMs (Belfi et al., 2022; Pearson et al., 2023). The LIWC data derived from the final category and subcategory (perception and cognition) reflect the percentage of total words devoted to each category in the description of the familiar musical section.

## 3 Results

### 3.1 Greater signal differences in the familiar listening condition

When listening to familiar compared to unfamiliar music, greater fALFF activation patterns were observed in the right parahippocampal gyrus (PHG), right angular gyrus, bilateral medial temporal gyri, and right visual association area at a 0.005 (uncorrected) voxel wise threshold. A 7-mm spherical small volume correction was applied to the *a priori* regions associated with autobiographical memory, specifically the hippocampus as well as parahippocampal and retrosplenial regions. After the small volume correction, significantly greater fALFF activation was observed in the right parahippocampal cortex (*k* = 20, *p* = 0.001 (uncorrected), *t* = 5.28, MNI = 21, −27, −6).

### 3.2 Greater signal differences in the familiar listening condition controlling for age and GMV

**Figure 1:**
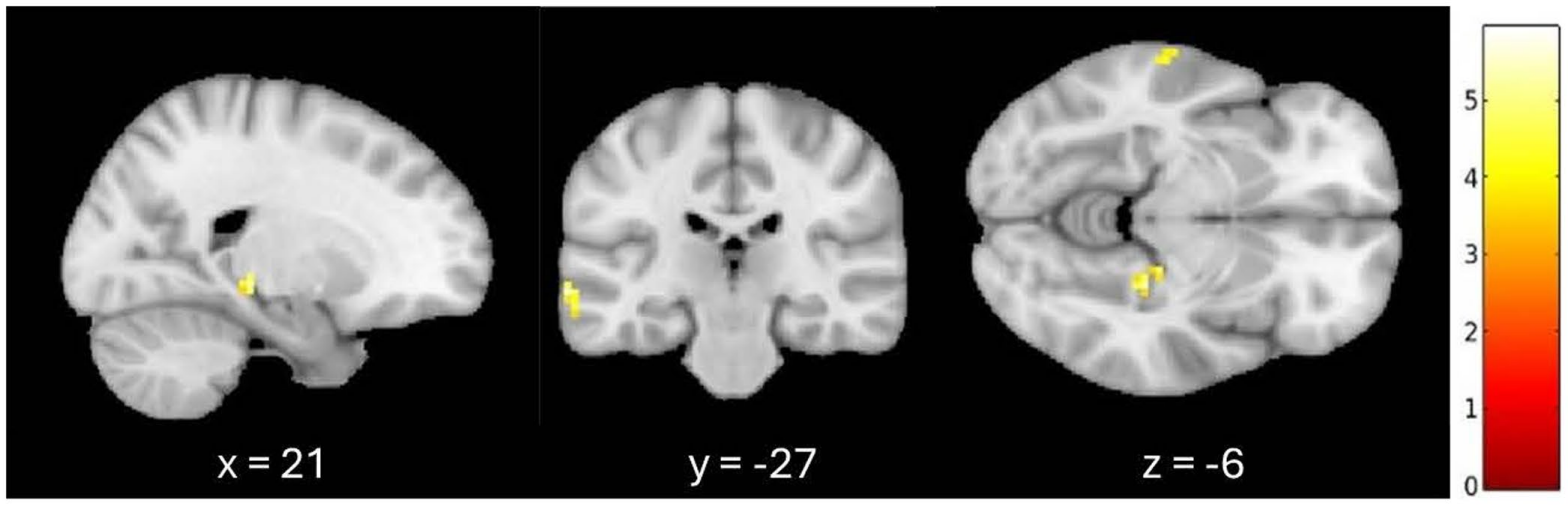
Greater signal differences in familiar versus unfamiliar listening conditions controlling for age and gray matter volume. Greater fALFF activation observed in the right parahippocampal cortex (MNI coordinates: *x* = 21, *y* = −27, *z* = −6; 6 voxels, *p* = 0.001, uncorrected).

An additional exploratory analysis was conducted to confirm these results when controlling for the effect of age and whole-brain GMV on fALFF. When listening to familiar compared to unfamiliar music, greater fALFF activation patterns were observed in the left medial temporal gyrus and the right parahippocampal cortex at a 0.001 (uncorrected) threshold, controlling for age and GMV. A 7-mm spherical small volume correction was applied to the *a priori* regions associated with autobiographical memory, specifically the hippocampus, parahippocampal cortex, and retrosplenial regions. After the small volume correction, significantly greater fALFF activation was observed in the right parahippocampal cortex (*k* = 6, *p* = 0.001 (uncorrected), *t* = 5.28, MNI = 21, −27, −6) when controlling for age and GMV (see Figure 1).

### 3.3 Greater signal differences in the unfamiliar listening condition

When listening to unfamiliar compared to familiar music, greater fALFF activation patterns were observed in the right premotor and supplementary motor areas (*k* = 446, *p* = 0.009 (uncorrected), *t* = 7.17, MNI = 9, 24, 57), left visuo-motor cortex (*k* = 223, *p* = 0.037 (uncorrected), *t* = 6.58, MNI = −27, −42, 66), and right visuo-motor cortex (*k* = 105, *p* = 0.077 (uncorrected), *t* = 6.26, MNI = 42, −51, 57). These areas showed significance using a *p* < 0.001 (uncorrected) threshold at the voxel level and *p* < 0.05 (uncorrected) threshold at the cluster level.

### 3.4 Greater signal differences in the unfamiliar listening condition controlling for age and GMV

An additional exploratory analysis was conducted to confirm these results when controlling for the effect of age and whole-brain GMV on fALFF. When listening to unfamiliar compared to familiar music, greater fALFF activation patterns were again observed in the right premotor and supplementary motor areas (*k* = 419, *p* = 0.018 (uncorrected), *t* = 5.03, MNI = 9, 24, 57), left visuo-motor cortex (*k* = 223, *p* = 0.052 (uncorrected), *t* = 4.81, MNI = −27, −42, 66), and right visuo-motor cortex (*k* = 33, *p* = 0.126 (uncorrected), *t* = 4.64, MNI = 42, −51, 57). These areas showed significance using a p < 0.001 (uncorrected) threshold at the voxel level and *p* < 0.05 (uncorrected) threshold at the cluster level. A 7-mm spherical small volume correction was applied to the *a priori* regions associated with autobiographical memory, specifically the hippocampus, parahippocampal cortex, and retrosplenial regions. After the small volume correction, no significant fALFF activation was observed in the *a priori* regions.

### 3.5 Correlations between age, fALFF, and whole-brain and regional GMV

**Figure 2:**
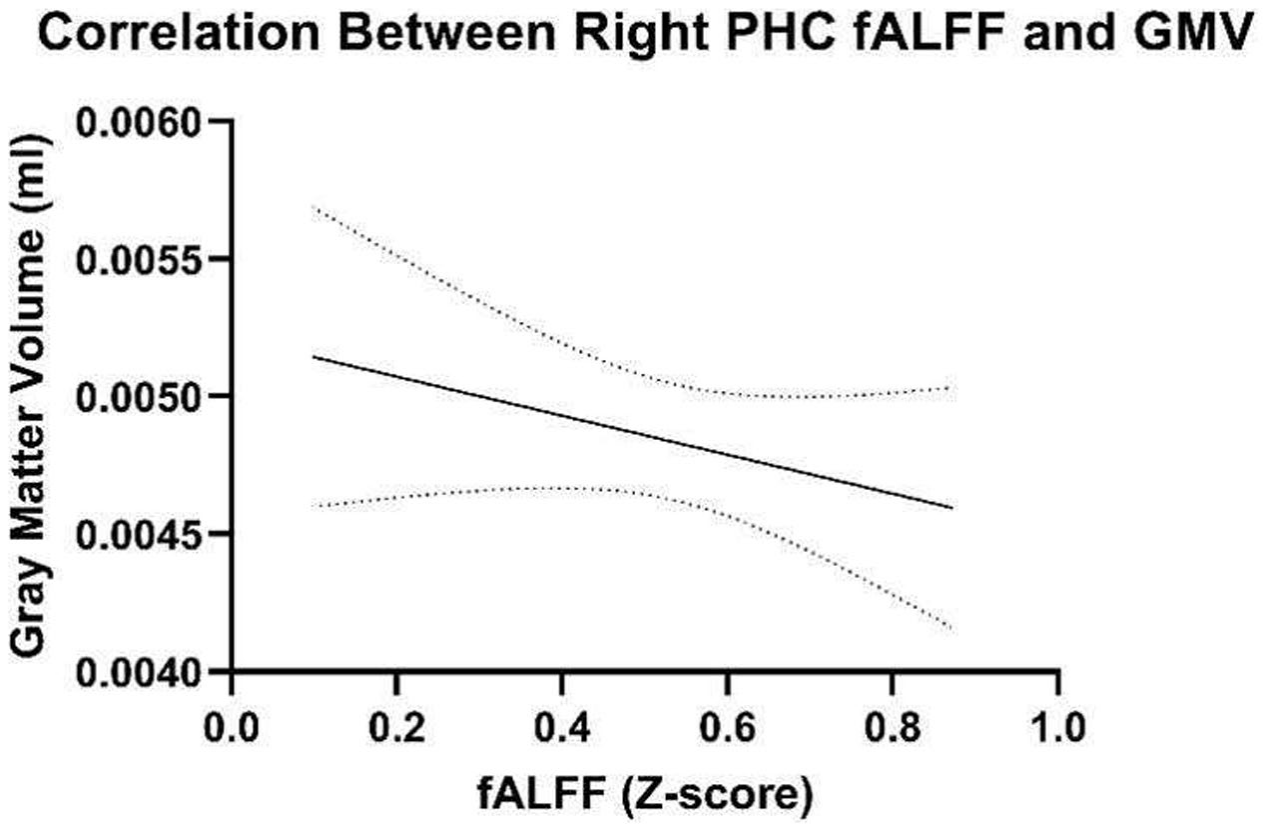
Correlations between fALFF and gray matter volume of the right parahippocampal gyrus.

Additionally, correlations were examined to determine the relationship between GMV of the right parahippocampal cortex and right parahippocampal fALFF during familiar listening. The right parahippocampal GMV and fALFF during familiar listening were not significantly correlated, *r* = −0.265, *p* = 0.106 (Figure 2). The whole-brain GMV was also not associated with right parahippocampal fALFF during familiar listening, *r* = −0.202, *p* = 0.345. This suggests that fALFF activation occurs independently of GMV. Additionally, as expected, across the sample, age and total GMV were negatively correlated (*r* = −0.361, *p* = 0.042).

### 3.6 *Post-hoc* analyses: brain activity and content of autobiographical memories

**Figure 3:**
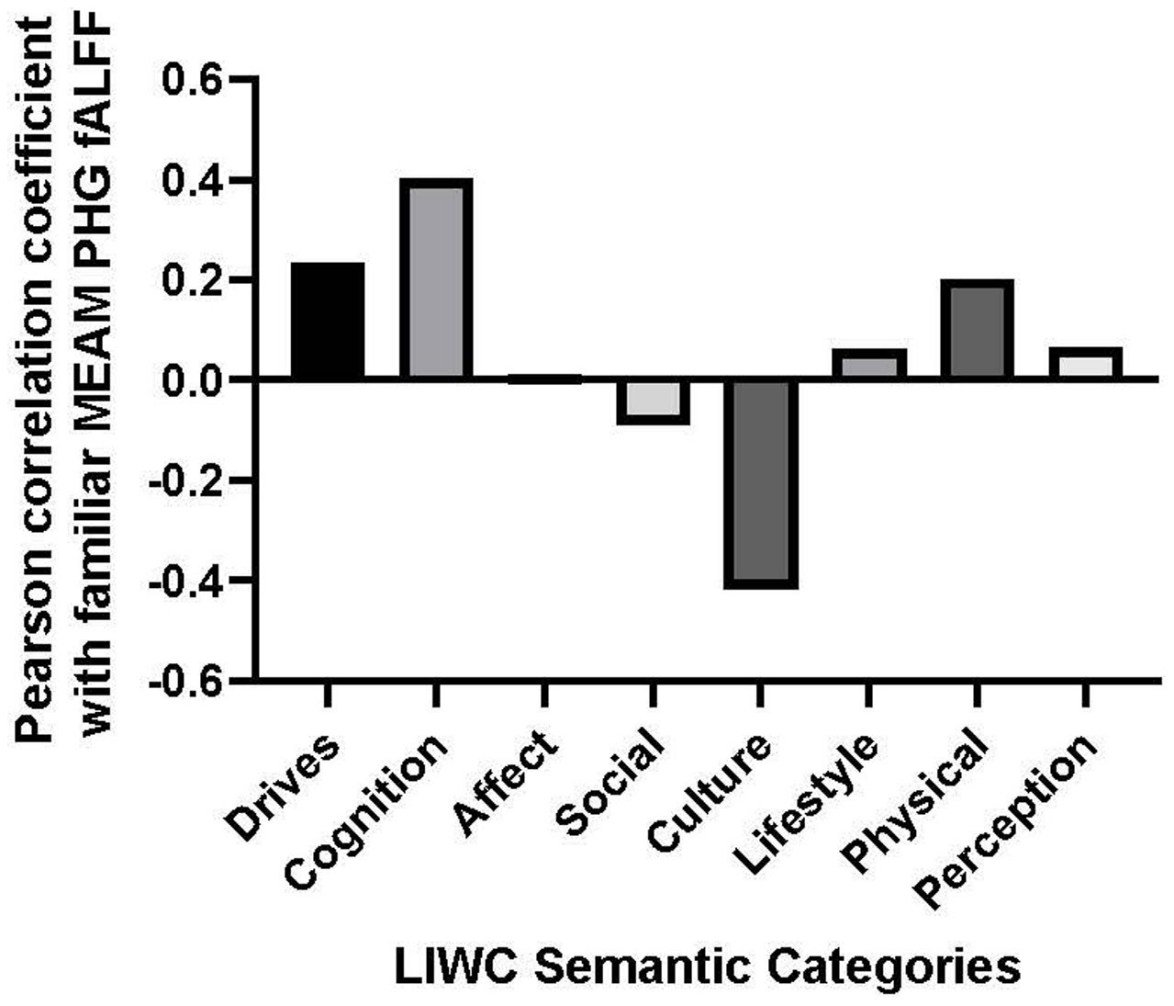
Bar graph of correlations between music-evoked autobiographical memory–elicited fALFF of parahippocampal gyrus and semantic categories from the Linguistic Inquiry and Word Count analysis.

*Post-hoc* analyses explored correlations between brain activity and the following dimensions of MEAMs: Drives, Cognition, Affect, Social, Cultural, Lifestyle, Physical, and Perceptions. Correlations between these supraordinate semantic categories and the mean parahippocampal gyrus fALFF during the familiar listening condition are plotted on a line graph (see Figure 3). A Bonferroni adjustment was made to the results of the two-tailed Pearson correlation coefficient test comparing fALFF to the aforementioned MEAM dimensions (0.05 ÷ 8 = 0.0625).

**Figure 4:**
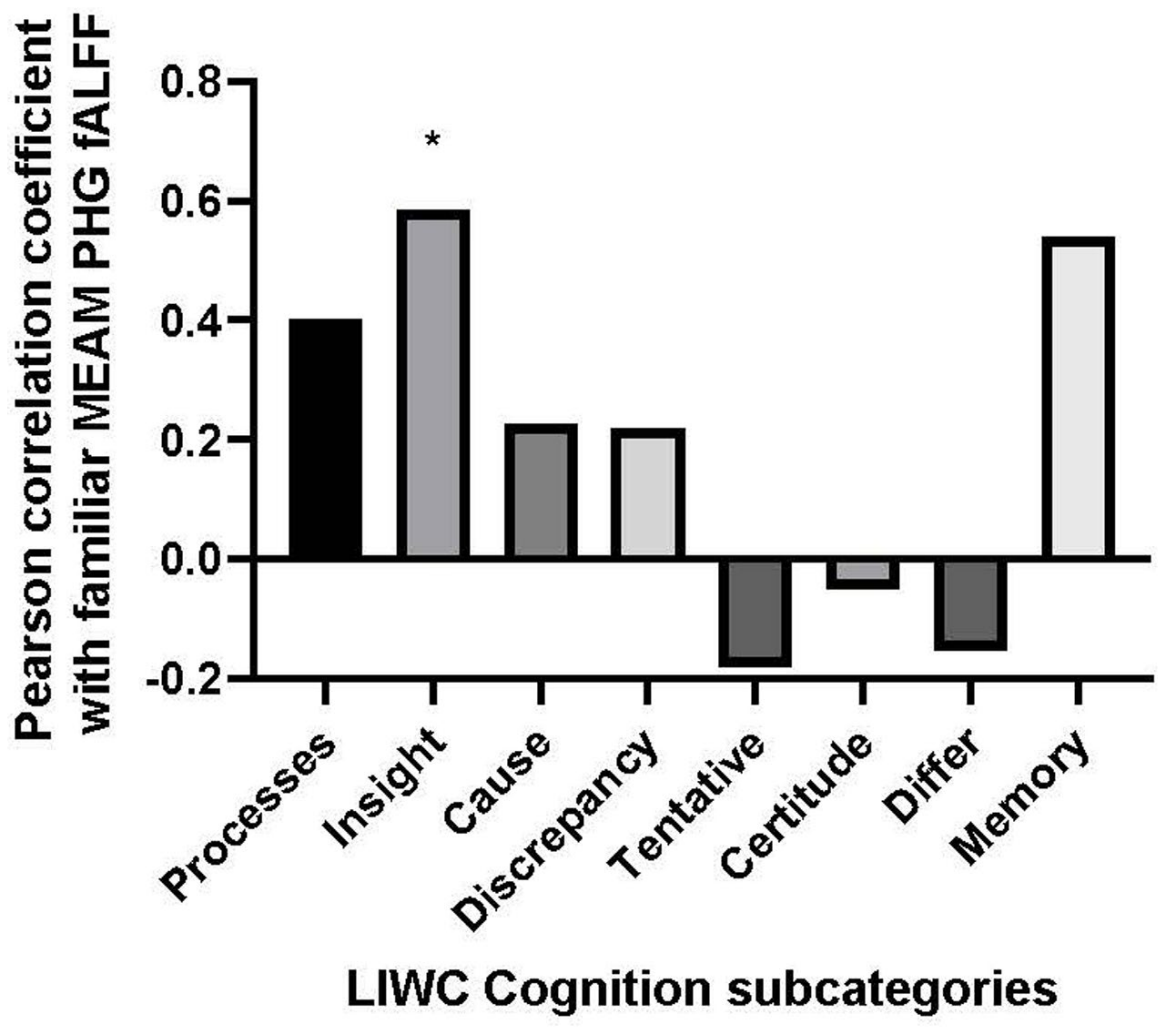
Bar graph of correlations between music-evoked autobiographical memory–elicited fALFF of parahippocampal gyrus and cognitive subcategories from the Linguistic Inquiry and Word Count analysis.

The only positive correlation between the semantic categories and bilateral fALFF observed at the trend level was with Cognition (*r* = 0.403, *p* = 0.057). Additionally, there was a negative correlation with the use of Culture words in describing the familiar musical selections, (*r* = −0.417, *p* = 0.048). However, the results of the correlational analysis did not meet the threshold of statistical significance after adjusting for multiple comparisons (*α* = 0.00625). Nevertheless, we explored the correlation between the PHG fALFF and the 8 subordinate LIWC Cognition categories based upon our *a priori* hypothesis that the cognitive LIWC category would relate to the hippocampal fALFF activation during the familiar MEAM. The categories included Cognitive processes, Insight, Causal, Discrepancy, Tentative, Certitude, Differ, and Memory (see Figure 4). Positive correlations and trends were observed between bilateral PHG fALFF and Cognitive processes (*r* = 0.402, *p* = 0.057), Insight (*r* = 0.585, *p* = 0.003), and Memory (*r* = 0.540, *p* = 0.008). After adjusting for multiple comparisons (*α* = 0.006), Insight was the only significant subordinate cognitive category to correlate with familiar parahippocampal fALFF (*α* = 0.00625).

## 4 Discussion

Previous research has identified several brain areas involved in MEAMs, but results have been inconsistent due to variations in methodological approaches. Differences in memory evocation techniques and the use of resting-state versus task-based analyses have led to mixed results in defining a clear neural signature for MEAMs. This study aimed to clarify which specific brain regions are activated during MEAM retrieval and to explore how these activations vary depending on the content of the memories. To address the methodological challenges observed in prior research, we used fALFF to measure brain activity in healthy older adults as they listened to familiar and unfamiliar music. We also examined how these activations are influenced by normal age-related changes in the brain structure, specifically GMV reduction.

Findings revealed that the right parahippocampal cortex exhibited greater fALFF when participants listened to familiar music than unfamiliar music. This result partly aligns with previous research, including Leaver et al.’s (2009) seminal study, which demonstrated significantly greater activity in the PCC and parahippocampal cortex during familiar music listening. The lateralization of the effect for parahippocampal activation, favoring the right PHG in the current study, may reflect the influence of musical expertise and training on enhanced retrieval of memories tied to familiar music (Groussard et al., 2010). Similarly, Satoh et al. (2006) observed increased regional cerebral blood flow throughout the medial temporal lobe and the parahippocampal cortex when participants judged the familiarity of musical pieces. Overall, these results underscore the central role of the parahippocampal cortex in processing familiar music and its broader significance in memory-related functions. As a key region in the medial temporal lobe, the PHG is crucial for memory formation and associative learning (Aminoff and Tarr, 2015), playing a central role in spatial and non-spatial aspects of contextual processing, scene processing, navigation, and episodic memory.

Importantly, the observed activation patterns in the right parahippocampal cortex remained robust even after controlling for age and GMV. GMV is known to decrease with normal aging (Hafkemeijer et al., 2014), particularly in the hippocampus and associated areas. This decline is further exacerbated in conditions such as Alzheimer’s disease (AD) (Nobis et al., 2019). In our study, although age was associated with significantly lower GMV across the whole brain, it did not affect the volume of the right parahippocampal cortex. Moreover, right parahippocampal fALFF during the familiar listening condition was not correlated with the GMV of the whole brain or the PHG, suggesting that the functional activity observed is not dependent on structural changes.

These findings underscore the potential for using fALFF in conjunction with volumetric analyses to gain new insights into the neural correlates of brain aging. In addition, these findings have important implications for cognitive aging. The ability of older adults to retrieve memories through familiar music may remain intact despite age-related structural decline in brain regions. Although further studies are needed, particularly in clinical populations such as those with MCI or AD, our findings raise the possibility that MEAMs could be leveraged in therapeutic contexts to stimulate brain regions involved in memory retrieval. Indeed, prior research has shown that individuals with MCI or AD exhibit differences in neural activation during familiar music listening, suggesting that MEAMs may engage spared memory networks in these populations (Thaut et al., 2020).

In addition to examining brain activity, we analyzed the language participants used to describe their autobiographical music selections. The words people choose can reveal much about their traits, cognitions, and behaviors (Aramaki et al., 2016; Chung and Pennebaker, 2018). To explore these dimensions, we used the LIWC protocol (Pennebaker et al., 2003), which has been successfully integrated with the MEAM paradigm in previous research. For example, logistic regression models trained on the LIWC and autobiographical interview data have been able to differentiate between memories evoked by popular music versus familiar faces with above-average accuracy (Belfi et al., 2020). A subsequent study confirmed that MEAMs evoke more LIWC-measured perceptual details than picture-evoked autobiographical memories (Belfi et al., 2022). In addition, previous research has shown that writing down spontaneously evoked memories provides richer perceptual details than orally recording them (Pearson et al., 2023). This observation further validated our *post-hoc* comparison of written descriptions of autobiographical memories evoked by the familiar musical selections with the magnitude of fALFF observed during those conditions.

While our study provides valuable insights into the neural mechanisms of MEAMs in healthy older adults, several limitations must be acknowledged. First, the sample size was relatively small (*N* = 24), which may restrict the generalization of the findings to a broad population. Second, fALFF focuses on low-frequency fluctuations, which may miss higher-frequency activity that could be relevant to MEAMs in certain brain regions. Third, the correlations reported between the fALFF activity and linguistic dimensions were not corrected for multiple comparisons, which increases the likelihood of false positives. Fourth, while it was our intent to have participants listen to music that they had no or minimal associations with, and evidence suggests no neural differentiation in cross-cultural comprehension (Morrison et al., 2003), we may have inadvertently examined differences in music cultures. Finally, despite past evidence suggesting that BOLD responses may not be sensitive to cultural differences in music selections (Morrison et al., 2003), neuroimaging paradigms involving MEAMs have not effectively accounted for the host of acoustic features that are shown to reliably alter the BOLD response such as evinced by fullness, brightness, activity, timbral complexity, and pulse clarity (Toiviainen et al., 2014). Future studies will need to, at the minimum, control for the effect of these acoustic features on BOLD response to MEAMs. Additionally, our study focused on healthy older adults, limiting the generalizability of the findings to clinical populations. Finally, our study examined neural activity within a relatively long music listening session (i.e., two 12-min sessions) in which conditions required while in an fMRI can be demanding or uncomfortable for participants. For example, the noise of the fMRI may intrude on listeners and the tight bore of the machine may impact one psychologically. However, any of these influences on the results were minimalized by the counterbalanced design of comparing uninterrupted listening conditions. Moreover, although the effect of scanner noise on strict resting-state fMRI is not negligible, the hippocampal and parahippocampal regions reported in our current study are not among those regions reportedly implicated in the effect of bore conditions on the low-frequency signal (Rondinoni et al., 2013; Skouras et al., 2013).

In conclusion, our study provides preliminary evidence that listening to familiar music evokes greater activity in the right parahippocampal cortex compared to unfamiliar music and that this activity correlates with the use of insightful cognitive words in describing autobiographical memories. Although our findings suggest a link between MEAM-evoked fALFF in the PHG and word generation in the cognitive-insight domain, these results may be biased as the results for the semantic category of cognition did not withstand control for multiple comparisons across the eight LIWC semantic domains. Hence, the importance of cognitive insight in familiar MEAM responses within the parahippocampal cortex should not be overstated due to the exploratory nature of those analyses. These findings correspond with other human neuroimaging and neuropsychological work implicating the PHG, in conjunction with fronto-hippocampal network, in source memory attribution, i.e., insight (Henson, 2005; Kounios and Beeman, 2014; Mayes et al., 2007; Mitchell et al., 2005; Shen et al., 2013). Future studies will need to compare the MEAM-related medial temporal lobe fALFF activity in healthy older adults to that of individuals at risk for early-onset dementia or MCI. This study may help to elucidate the therapeutic potential of MEAMs.

## Data Availability

The raw data supporting the conclusions of this article will be made available by the authors, without undue reservation.

## Footnotes

^1^ http://rfmri.org/dpabi

^2^ http://www.fil.ion.ucl.ac.uk/spm
