# Supplementary material for: Fractional amplitude of low-frequency fluctuations during music-evoked autobiographical memories in neurotypical older adults
Source: Front Neurosci. 2025 Jan 23;18:1479150. doi: 10.3389/fnins.2024.1479150 (PMC11800146; doi:10.3389/fnins.2024.1479150)
Supplement: Supplementary file 1 [file Table_1.pdf]

Table S1a

Acoustic Parameters

Metric	Familiar		Unfamiliar		*t*-statistic	*p*-value
	Mean	*SD*	*Mean*	*SD*		
Flatness	0.07	0.02	0.10	0.001	-6.21	<.001
Brightness	0.50	0.08	0.64	0.001	-8.85	<.001
Pulse Clarity	0.31	0.15	0.23	0.001	2.61	0.016
Key Clarity	0.76	0.09	0.58	0.002	10.3	<.001
Loudness (RMS)	0.13	0.04	0.09	0.001	5.2	<.001

Table S1b

Acoustic Parameters and Music-Elicited ALFF

Metric	Familiar		Unfamiliar	
	Left PHC	Right PHC	Left PHC	Right PHC
Flatness	0.24	0.06	-0.06	0.12
Brightness	0.03	-0.08	-0.18	0.11
Pulse Clarity	0.02	-0.17	-0.33	-0.13
Key Clarity	-0.04	-0.09	-0.01	-0.03
Loudness (RMS)	0.30	0.13	-0.11	0.06
