# Supplementary material for: Fractional amplitude of low-frequency fluctuations during music-evoked autobiographical memories in neurotypical older adults
Source: Front Neurosci. 2025 Jan 23;18:1479150. doi: 10.3389/fnins.2024.1479150 (PMC11800146; doi:10.3389/fnins.2024.1479150)
Supplement: Supplementary file 2 [file Supplementary_file_1.pdf]

Figure S1

Research Phases

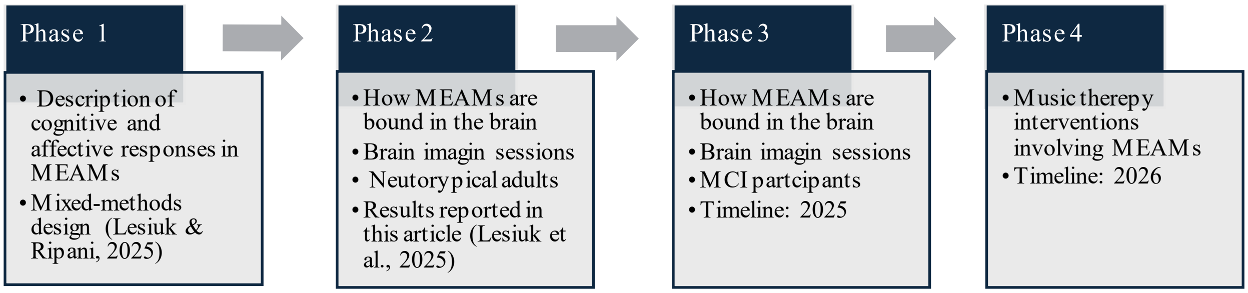

Note. MEAMs stands for music-evoked autobiographical memories. Figure S1 illustrates the larger research project, with this study representing the second phase.
