# Supplementary material for: Fractional amplitude of low-frequency fluctuations during music-evoked autobiographical memories in neurotypical older adults
Source: Front Neurosci. 2025 Jan 23;18:1479150. doi: 10.3389/fnins.2024.1479150 (PMC11800146; doi:10.3389/fnins.2024.1479150)
Supplement: Supplementary file 3 [file Supplementary_file_2.pdf]

Figure S2

Participants' Flow of Activities

First Questionnaire	Brain Imaging Sessions	Second Questionnaire	Phone Interviews
•On-line<br>•Questions on participants' responses to self-selected autobiographical music<br>•Format of questions: (a) with categorical answers, (b) open-ended, and (c) Likert-type	•In-person<br>•fMRI	•On-line<br>•Questions on participants' responses to autobiographical and non-autobiographical music during the fMRI sessions<br>•Format of questions: (a) with categorical answers, (b) open-ended, and (c) Likert-type	•Phone<br>•Up to 30 minutes<br>•Follow-up questions to gain more details on participants' responses to music during fMRI sessions

Note. Figure S2 summarizes the flow of activities that participants undertook in phases 1 and 2 (Figure S1) of our larger research project. The results of Questionnaires 1 and 2 and the phone interviews are described in Lesiuk & Ripani (2025). Results from Questionnaire 2 confirmed that, during the brain imaging sessions, all autobiographical excerpts elicited memories, while non-autobiographical pieces evoked either no associations or only loose associations. Additionally, participants reported that the non-autobiographical music was largely unfamiliar.

The fMRI protocol included a 12-minute resting state sequence, a T1 structural sequence (TR/TE = 2300/1 ms, FA = 9□, thickness = 1.0mm, matrix size = 256 x 256), 12-minute autobiographical (familiar) music listening, arterial spin labeling, 12-minute unfamiliar music listening, and diffusion-weighted imaging. The familiar and unfamiliar music listening tasks were presented in a randomized order.

Autobiographical music excerpts were randomly selected among participants' self-selected pieces. Participants predominantly selected music from the rock and pop genres, with songs mainly from the 1970s and 1980s, reflecting their late adolescence and early adulthood. We created the non-autobiographical musical excerpts by combining three pieces that do not belong to common Western repertoires (i.e., Balinese Gamelan, Mongolian throat singing, and Zimbabwean Mbira music), as recommended by Dr. Donald Hodges (personal communication).

Link to Music for Non-Autobiographical Excerpts

Balinese Gamelan

Mongolian Throat Singing

Zimbabwean Mbira Music
